# Observations on the Structure and Disease of the Testis

**Published:** 1831-04-01

**Authors:** 


**OBSERVATIONS ON THE STRUCTURE AND DISEASES OF THE TESTIS. By Sir *Astley Cooper*, Bart. F.R.S. Serjeant Surgeon to the King, Consulting Surgeon to Guy's Hospital, &c. &c. &c. Longman's London. 1830.**

[Third and concluding Article.]

WE have already dedicated two ample, most ample, articles to the splendid and valuable work of Sir Astley Cooper, the title of which is seen above. If those articles have run to an almost unprecedented length, they have not exceeded their just dimensions by a single line. We do not pretend that Sir Astley Cooper is always right, or that his opinions are infallible; for such attributes are not of mortality. But we do affirm, and we conscientiously believe it, that practical men may rely upon the facts and the inferences contained in the work in question, and that all unprejudiced persons will acknowledge it a boon to the profession. It consists, as we have said on a former occasion, of two distinct parts; the one descriptive of the anatomy of the testis, the other of its diseases. We have considered the latter very fully, and it now only remains for us to direct our readers' attention to the former. The anatomy of the testis! Some gentlemen, fresh perchance from the lectures and the schools, may wonder what Sir Astley can say upon that. Older practitioners may smile at such a subject, and turn away with a shrug from a thing so barren in practical interest. To the former we shall have a sufficient answer bye-and-bye. To the latter we may as well say a word or two at once. Unless the healthy and natural structure of an organ be perfectly understood, its morbid alterations, organic or functional, can never be satisfactorily or scientifically investigated. Would a man send his watch to be repaired by one who knew not perfectly its nature and construction! Surely he would not. Neither can a practitioner pretend to an honourable rank in his profession, or lay a just claim to the character of a man of science, unless he be acquainted with the structure of the organ, the diseases of which he attempts to control.

The question of direct advantage, the *cui bono*, from any particular department of knowledge, is one which irresistibly presents itself to the minds of the mass of mankind. In this money-getting, money-loving country, it is the catechism of the creed of the man of the world. Yet it cannot but be owned, that if every investigation were at once to be judged upon such a query, we should now be deficient in many of the most splendid, aye, and useful, of our modern improvements. We cannot forbear from quoting a passage, bearing on this subject, from the recent production of a very able and scientific gentleman; we allude to Mr. Herschel.

"If," says Mr. Herschel, "the natural philosopher can bring himself to descend from this high but fair ground, and justify himself, his pursuits, and his pleasures in the eyes of those around him, he has only to point to the history of all science, where speculations apparently the most unprofitable have almost invariably been those from which the greatest practical applications have emanated. What, for instance, could be apparently more unprofitable, than the dry speculations of the ancient geometers on the properties of the conic sections, or than the dreams of Kepler, (as they would naturally appear to his contemporaries,) about the numerical harmonies of the universe? Yet these are the steps by which we have risen to a knowledge of the elliptic motions of the planets and the law of gravitation, with all its splendid theoretical consequences, and its inestimable practical results. The ridicule attached in Hooke's time to 'swing swangs' did not prevent him from reviving the proposal of the pendulum as a standard of measure, since so admirably wrought into practice by the genius and perseverance of Captain Kater;—nor did that which Boyle encountered in his researches on the elasticity and pressure of the air act as any obstacle to the train of discovery which terminated in the steam-engine. The dreams of the alchymists led them on in the path of experiment, and drew attention to the wonders of chemistry, while they brought their advocates (it must be admitted) to merited contempt and ruin. But in this case it was moral dereliction which gave to ridicule a weight and power not necessarily belonging to it; but among the alchymists there were men of superior minds, who reasoned while they worked, and who, not content to grope always in the dark, and blunder on their object, sought carefully in the observed nature of their agents for guides in their pursuits; to these we owe the creation of experimental philosophy."

We think we need pursue this subject no farther. A strict investigation of anatomical facts, a rigid enquiry into the intimate structure of parts should form the basis of all medical reasoning and observation; and, although our art must be always in a degree empiric and conjectural, yet such a method of research bids fair to redeem it from some of its uncertainty.

Sir Astley Cooper is one of those men whose love of truth makes them doubt what they do not see. From his converse with the world he knows the prevalence of unconscious error, and the boldness of wilful falsehood. He doubts what he reads in books, and, for ought that we know, such doubts may be aright. Yet, *all* that is printed cannot be untrue, or the world would not be under so many obligations to Sir Astley for his own practical writings. We cannot refrain from mentioning a circumstance, although unauthorized to do so, which reflects great honour on the distinguished individual to whom we are alluding. At a time when he was reputed as enjoying the luxurious repose of an English country seat, and the healthsome amusements of the field, Sir Astley Cooper was busily employed in making the preparations upon which the anatomical descriptions of his work are founded. Those preparations are at present in Sir Astley's possession, and more splendid specimens of the art of the anatomist have never been witnessed since the days of Ruysh. With the urbanity that has always peculiarly distinguished him, Sir Astley is ever ready to display them to all who take an interest in the subject. With these few remarks we proceed to the work of analysis.

Passing over the description of the vessels and nerves of the scrotum, we halt at the DARTOS. According to Sir Astley this exists only in the imagination of the anatomist. The motions of the scrotum are not muscular and sudden contractions, but vermicular and gradual; they are not voluntary; they result from changes of temperature; and seem to depend on the lessened diameters of the arteries and veins of the part, and of the diminished quantity of blood which they contain.

*Cellular Tissue of the Scrotum.* This is loose, long, reticular, connecting the integuments to the external covering of the spermatic cord and testis.

It is rather reticular than adipose, to prevent any increase of bulk under corpulency; long and loose, to permit great freedom of motion in the testis, and enable it to elude the influence of violence. Opposite the raphé, it becomes condensed, and is named the septum scroti, although it is permeable to air and water, allowing dropsical effusion to pass through it, and the whole reticular membrane to be distended. From the septum, reticular fibres pass to the covering of the testis, to preserve each in its situation. The septum is supplied with blood from the perineal artery, which anastomoses freely with the external pudic. When the diseased testis adheres to the septum, these vessels are greatly enlarged, and often furnish, if not secured, a troublesome hæmorrhage.

*Superficial Fascia of the Cord.* This fascia proceeds from the surface of the tendon of the external oblique, and the margin of the ring, envelops the cord and descends upon the testicle, which it covers. Internally, it is attached to the cremaster muscle and its tendon—externally, it is connected by reticular fibres to the cellular tissue of the scrotum.

*Cremaster Muscle.* This envelops the cord and covers the testis, but Sir Astley defers its description to another place.

*Tunica Vaginalis.* "This membrane, when first raised, is found to be covered entirely by the tendon of the cremaster muscle, which envelops its outer surface, and is inserted into it; and until this be cut through, the true tunica vaginalis does not appear.

When the insertion of the cremaster muscle is cut away, the tunica vaginalis is found to be a very delicate and thin membrane, formed from the peritonæum, and descending from the abdomen before the testis. It is composed of two portions: the one loose, and detached from the testis, excepting posteriorly and laterally; the other, which adheres to the surface of the tunica albuginea, and which covered the testis whilst in the abdomen; but when examined in the scrotum, the two portions are connected, and are continuations of each other.

The first, or loose portion is the tunica vaginalis reflexa, and the adhering portion the tunica vaginalis testis: between the first and second there is a cavity, into which a vapour, or halitus, is naturally secreted, and which, when poured out in a diseased quantity, produces the complaint which is called hydrocele.

The tunica vaginalis is a reflected membrane, like the pericardium, pleura, and peritonæum. The tunica vaginalis reflexa passes loosely over the fore-part and sides of the testis; and being continued to its posterior edge, there turns over the epididymis to the surface of the testicle, covering and adhering to the tunica albuginea; and in a similar manner on the inner side, excepting on that side there is no epididymis.

The tunica vaginalis testis can be dissected from the tunica albuginea but to a short distance, as it soon becomes incorporated with the surface of that membrane.

Behind the tunica vaginalis reflexa, and the tunica vaginalis testis, the testicle is placed, contained in its tunica albuginea; and the spermatic vessels, the vas deferens, the absorbents, and the nerves of the testicle enter it posteriorly, and do not penetrate the tunica vaginalis; and the testis may be cut into behind without injury to that tunic.

In this dissection, then, the scrotum is first cut through; next the cellular tissue; thirdly, the fascia superficialis; fourthly, the cremaster muscle; fifthly, the tunica vaginalis reflexa; sixthly, the tunica vaginalis testis; and then the testis, with its covering of tunica albuginea, is exposed.

The tunica vaginalis is a serous membrane, and forms a cavity which communicates with the peritonæum and cavity of the abdomen before birth, but is usually shut after birth by adhesion, when it becomes a small thin cord, situated on the fore-part of the spermatic vessels. The fluid which it secretes, when abundant, has the colour and other properties of serum, being a solution of albumen. It is coagulable by heat, and various chemical agents. The tunic is supplied with vessels from the spermatic artery, and artery of the vas deferens, from which its halitus is secreted. Its veins open into the spermatic veins. Its absorbents pass upon the spermatic cord with those of the testis, and with them into the abdomen; and its nerves are in part derived from the spermatic plexus, and in part from a branch of the external spermatic. It possesses considerable sensibility, and irritation of it produces sickness. In the healthy state, when opened, no fluid is found in it; but a vapour arises, and it becomes dry.

When the tunica vaginalis reflexa is opened, the cavity which is situated between it and the tunica vaginalis testis, is exposed; and through the latter membrane, which is semi-transparent, the tunica albuginea testis appears. The general form of the testis and epididymis may be observed, the latter being placed upon the upper, posterior, and outer part of the testis; beside which a little vascular membranous body is also seen upon the anterior extremity of the caput epididymis." 12.

*Of the Testis.* This is oviform, situated rather obliquely, with its largest extremity placed upwards and forwards. It has an anterior and superior, a posterior and inferior extremity; an anterior and inferior, a posterior and superior edge; and it has two lateral surfaces. Its anterior edge is most rounded, the posterior least so; the two sides are convex, although flatter than the anterior edge. At the posterior edge the spermatic vessels enter, and this part is devoid of the tunica vaginalis. The upper extremity of the testis is capped by the epididymis. The axes of the testis are three. The longest, which is two inches in a healthy formed testis, passes from the anterior and upper extremity to the posterior and lower; the second axis is one inch and a half, and passes from the posterior superior to the anterior inferior edge; the third or transverse diameter, passing from side to side, is one inch and one-eighth in length. The weight of a healthy testis and epididymis is about an ounce.

*Of the Tunica Albuginea Testis.* This forms a complete covering to the glandular structure of the testis, leaving a cavity in which it is contained; but at the upper and posterior part of the testis, a little to its outer side, the tunica albuginea turns in towards the centre of the testis, and forms a triangular process, which, from its situation, Sir Astley is disposed to call the mediastinum testis. This inverted portion of the tunic sends forth numerous ligamentous cords. Some pass directly from the mediastinum to the anterior edge of the testis, and form pillars, which are strongly fixed to the inner side of the tunica albuginea, to prevent the separation of its sides; others, more numerous, but smaller, descend upon the seminiferous tubes, and send forth lateral membranes, which form purses to enclose the lobes into which the glandular structure is divided: and these are met by similar ligamentous cords and membranes, from the inner surface of the tunica albuginea, to complete the envelope of the lobes of the testis.

The tunica albuginea is, therefore, not merely a simple bag to enclose the glandular structure of the testis, but it forms a process which splits into ligamentous cords; and these send forth lateral membranes, which divide the glandular structure into lobes, in which the seminal tubes are contained. The membranes and cords not only support and connect the seminal tubes, but form beds upon which arteries, veins, absorbent vessels, and nerves are spread. They have been called septa: but in reality they envelop the seminiferous tubes, convey to them the blood, and form bags which support, confine, protect, and nourish the tubular structure of the testis.

The outer surface of the tunica albuginea is covered by the tunica vaginalis testis, which is thin, soon incorporated with its surface, and only separable from it to a small extent. As it is a serous membrane, it renders the outer part of the tunica albuginea a secreting surface. The tunica albuginea is further divisible into two layers. The outer is fibrous, tendinous, inelastic, resembling the sclerotica and external portion of the dura mater. It is little vascular; but strong and inelastic to protect the tender tubular structure of the testis from violence, which it generally does to a surprising degree. The inner coat or layer of the tunica vaginalis is called by Sir Astley the tunica vasculosa; as the spermatic artery ramifies in it. It adheres pretty firmly to the outer layer at the anterior edge of the testis, where some of the internal ligamentous cords are fixed. But it may be entirely dissected from it, so as to form a separate preparation, enclosing the tubuli, and leaving the outer layer with the spermatic cord. This vascular tunic is easily demonstrated by filling the arteries and veins with fine injection; and then cutting open the testis and removing the tubuli. The membrane is seen highly vascular on the inner part of the tunica albuginea.

The outer layer resembles the dura mater in structure, and like it, forms processes internally. The inner membrane resembles the pia mater, being reflected inwards on the lobes of the testis, forming a bed on which the branches of the spermatic artery ramify, and supplying with vessels the membranes which envelop the tubuli. The arteries which supply the tunica vasculosa pass between this coat and the proper albuginea, before they divide into minute branches, to supply the membrane which is reflected inwards. Some branches of the spermatic veins also ramify upon the surface of this membrane; but the greater number pass, upon the ligamentous cords, into the glandular substance of the interior of the testis. Upon this membrane absorbent vessels are also found.

*Lobes of the Testis.* The tubuli seminiferi are disposed in numerous lobes, which are contained in the tunica albuginea; had they been loosely suspended in it they would have constantly been liable to derangement from concussion, or to laceration. The lobes are pyriform; their stalk, or commencement, is turned to the upper and posterior edge of the testis, their bases to the anterior and lateral parts of the tunica albuginea. They receive suspensory cords or ligaments from the mediastinum testis, which send out membranes to be spread over the lobes, and which meet others springing from the anterior edge and sides of the testis. Thus the lobes are confined and suspended by the ligaments and membranes.

*Of the Tubuli Seminiferi.* The cavity formed by the tunica albuginea is in great measure filled by the tubuli seminiferi, divided into two sets of lobes; first, into large lobes, enveloped in membranes and connected with the larger ligaments or pillars of the testis: and secondly, into an infinite number of small lobes, each also contained within a membrane. The larger are formed of numerous tubuli clustered together; the smaller of a single tubulus, and sometimes of two tubuli. The larger are pyriform, their stalks attached to the rete, their bases to the inner side of the tunica albuginea; they are situated between the stronger pillars of the ligaments of the testis, as they pass from the mediastinum to the inner part of the tunica albuginea; and the vascular membranes, by which they are enveloped, pass from one ligament to the other. The smaller lobes are also disposed in vascular membranes, and supported by smaller ligaments and vessels. Each tubulus begins from one of the canals which form the rete; and, passing through a small hole in the mediastinum testis, it becomes excessively convoluted, and forms a conical or pyriform body, the basis of which is turned to the inner side of the tunica albuginea, and the convolutions are placed nearly at right angles with the long axis of the tubulus. Each tubulus may be unravelled, when it is found to be composed of a long, single, and convoluted vessel, the convolutions disposed nearly in parallel lines, and nearly transversely to the long axis of the lobe. With these tubuli, thrown into larger and smaller lobes, and supported by ligaments from the mediastinum, is the cavity of the tunica albuginea filled.

The following blood-vessels are distributed upon the lobes. The *spermatic artery* passes in two large branches on the opposite side of the testis to the epididymis; and, between the outer and inner layer of the tunica albuginea, they are continued upon the inner coat towards the anterior and inferior edge of the testis. They there form an arch of communication, from which vessels pass upwards and backwards upon the membranes which cover the lobes of the tubuli. When they have reached two-thirds of the way to the mediastinum, they divide into two branches, which turn back on each side towards the anterior edge, and supply the membrane abundantly with vessels. The smaller lobes receive a little vessel at each extremity. The principal branches of the *spermatic veins* are arranged in a different manner. A few pass on each side on the surface of the lobes, but the greater number descend on the mediastinum, and are continued on the ligaments of the testis, between the larger lobes, to the anterior edge, when they become inverted, to be distributed upon the extremities of the larger lobes; they also meet some small veins which pass in at the anterior edge, and are distributed upon the extremities of the lobes.

*Of the Rete.* By this is meant a set of canals which receive the serum from the tubuli; not placed, like them, in the cavity of the tunica albuginea, but situated between its layers, in what Sir Astley has called the mediastinum. This is placed at the posterior edge of the testis, exactly opposite to the epididymis.

"To dissect this structure clearly and distinctly, first make a transverse section of the testis, and then, looking at its divided edge, it will be seen that the tunica albuginea is at that part readily divisible into three layers. The first layer turns upon the spermatic cord, uniting with the sheath which covers its vessels. The second layer unites with a similar layer on the opposite side, and forms a thick substance, between the fibres of which, interstices are left for blood-vessels and absorbents; whilst the internal layer, uniting with that on the opposite side, as well as with the preceding layer of the tunica albuginea, forms the process which I have called mediastinum, which projects into the testis between the tubuli; and it is in this substance that the seminal canals of the rete are placed. The mediastinum is therefore composed of two bodies—the upper placed towards the spermatic cord, the lower towards the centre of the testis:—in the upper are situated blood-vessels; in the lower, the canals of the rete; and from the lower proceed the pillars which are stretched to the inner side of the tunica albuginea, to bind its sides together, and smaller ligaments are also sent to the lobes of the tubuli, to envelop and support them." 20.

If an incision be made in the long axis of the testis from one end to the other, the mediastinum is seen projecting downwards and forwards amidst the tubuli, reaching more than three-fourths of the length of the testis, and its edge terminating in its ligaments. In the whole length of the mediastinum the canals of the rete are passing. In a transverse section of the mediastinum, these canals are very visible to the naked eye: passing in a longitudinal and waved direction from the posterior to the anterior part of the mediastinum, and situated in it more to the anterior and lower than to the posterior edge of the testis. Hence the mediastinum is composed of two parts: the back part of blood-vessels, the anterior of the rete. In these canals the tubuli terminate by single vessels, which pass through small apertures between the ligaments of the mediastinum, and they enter the anterior edge, as well as the sides and extremities of the rete; but they do not enter the posterior edge of the mediastinum. The rete terminates at the upper and posterior extremity of the testis, by forming the vasa efferentia. The mediastinum descends towards the centre of the testis, and the central tubuli there enter it, whilst the others pass into its sides. The back part of the mediastinum has a very convoluted artery, passing from one extremity to the other. The veins also ramify upon the back of the mediastinum, and send vessels through it, which pass between the ligaments and lobes of the tubuli.

"Having traced the canals of the rete, and found that they were situated in, and completely enclosed in the tunica albuginea, it struck me that I might inject these tubes with glue, or even coarse injection, by passing a fine silver or steel pipe into the canals of the rete; and having made trial of this plan, I have injected the tubuli seminiferi with coloured fine injection, and the vasa efferentia were also readily filled, and have been thus able to make some beautiful preparations, more easily dissected, and much less easily spoiled, than those which are made by injecting the tubes with quicksilver. The rete can even be filled with coarse injection; and the beginnings of the tubuli and the vasa efferentia will receive the injection. If the injecting pipe be placed in the back of the mediastinum, the injection readily escapes into the absorbent vessels, and those of the spermatic cord become filled." 22.

*Of the Vasa Efferentia.* The tubuli and the rete, already described, compose the body of the testis. The seminal vessels next in order are the vasa efferentia. These canals are placed between the testis and epididymis, and form the medium of communication between them. The greatest number which Sir A. has seen is fifteen, and from thirteen to fifteen exist in a healthy testis. But they are often found obliterated and reduced by disease to the number of six or seven, which does not prevent the organ from continuing to perform its functions, or hinder the conveyance of the semen through the remaining channels into the epididymis. The vasa efferentia arise singly from the anterior and upper extremity of the rete, and they terminate in different parts of the epididymis, so as to leave it a single tube. Prior to their termination, they each form a conical body, in which the seminal tube is divided with extreme minuteness. A small band of communication is continued along the surface of the vasa efferentia, to receive the termination of those vessels. Between the vasa efferentia and the lobes which they form, there are strong ligamentous cords, strengthening the connexion between the testis and epididymis; and the tunica vaginalis reflected over them is denser than in other parts. The vasa efferentia have the general form and character of the tubuli testis, save that their direction is reversed. They begin from the rete in single vessels, a little convoluted, and then by their excessive convolutions, they are formed into conical bodies; they differ from the tubuli in sending forth a vessel to the epididymis, instead of terminating in a blind extremity. The first vas efferens has the readiest communication with the epididymis, the second a smaller, and so on, although all ultimately communicate with it.

### OF THE EPIDIDYMIS.

This may be considered as an appendix to the testis. It is of crescentic form; its upper edge rounded; its lower edge thin. Its anterior and upper extremity is called its caput, the middle part its body, and the lower part its cauda. The caput and cauda have been called globus major and minor, but there is no enlargement entitled to the name of the latter. The epididymis is covered by the tunica vaginalis reflexa. The tunica vaginalis testis passes from the side of the latter directly over the caput and cauda of the epididymis; but in the centre it passes under the body of the epididymis to the spermatic cord; then turns, and lines the inner side of the epididymis, and rises over its sharp edge, to cover the upper part of its body, being continued to form the tunica vaginalis reflexa. It covers the cauda superficially; it closely invests the caput; and thus each extremity of the epididymis is confined to the testis, whilst, at its centre there is a hollow between the two, into which the point of the finger may be passed, and which is lined by the tunica vaginalis. When the tunica vaginalis is raised from the epididymis, numerous cords and branches of blood-vessels may be observed passing into it from the posterior to the anterior extremity, dividing it into lobes. These cords are the insertions of the cremaster muscles into the epididymis, and they also form bands, which prevent the convolutions of the tubes from being displaced.

*Of the Caput Epididymis, or Globus Major.* This is principally formed of the lobes of the vasa efferentia, sometimes named coni vasculosi, which are not situated in a single line, but some are placed behind others; so that the end of the epididymis is curved and double. Between these lobes are tendinous cords, which separate and support them; and on the upper part of the vasa efferentia a band of epididymis passes, which receives a vessel from each lobe of the vasa efferentia.

*Of the Cauda Epididymis.* Its cauda terminates in the vas deferens, the tube of which is larger and less convoluted than that of the epididymis, which is their chief distinction. In injecting the testicle, the quicksilver passes with difficulty from the vas deferens into the epididymis, from the sudden turn the tube here makes, and from its being bound down by cords proceeding from the cremaster.

*Of the Body of the Epididymis.* This is entirely composed of the convolutions of a single seminal tube, thrown into lobes, which convolutions pass in parallel lines from edge to edge. That it is a single tube, is shewn by its admitting of being entirely unravelled after maceration. It is subject to some varieties. Sir Astley has seen it naturally unravelled in its centre, to the extent of three quarters of an inch; and, secondly, it very frequently sends forth an additional vas deferens, from one to three inches in length, along the spermatic cord. Sir A. has a preparation of three of these in the same testicle, each terminating in a blind extremity. The arteries and veins have been already described; the absorbents of the epididymis pass into those of the spermatic cord, about an inch from the convex edge of the crescent.

### Of the Vas Deferens.

This begins from the cauda epididymis, and terminates in the duct of the vesicula seminalis, the combined vessels opening at the veru montanum in the prostatic part of the urethra. At its beginning from the epididymis it is doubled upon that body, and bound down by the tendinous fibres and insertions of the cremaster; it is here very much convoluted, though less so than the epididymis, and it does not form any distinct lobes. It descends below the cauda of the epididymis, at its commencement, and for the first inch its convolutions are numerous; in the second inch of its ascent, they become less in number; and in the third inch, from its beginning, in a great degree disappear. Its course we need scarcely describe. The vas deferens is enclosed in a sheath, formed by the tendinous fibres of the cremaster, and is supported by ligaments of its own which descend from the internal ring; the sheath may be readily found in the first three inches of the tube from the epididymis. The ligaments strengthen the connexion of the testis to the body and support it, and they preserve the convolutions of the vas deferens, for which its two lateral bands are particularly designed. It is situated posteriorly in the cord, there being a space of a quarter to half an inch between it and the spermatic artery and vein. Its roundness and hardness distinguish it. It is muscular in the bull, and its fibres take a circular direction. Between the vesiculæ, these vessels become enlarged, and their internal surfaces cellular, secreting a fluid which mixes with the semen. The structure of the vas deferens, near its termination, bears a strong resemblance to that of the vesiculæ seminales.

### Of the Spermatic Cord.

The parts composing it are situated in the abdomen, the inguinal canal, and between the abdominal ring and testicle. It consists of three arteries, with their corresponding veins, of the vas deferens, of absorbents, and of nerves covered by fascia, and by the cremaster muscle.

*Of the Parts in the Abdomen.* Without particularizing the origin and course of the spermatic arteries, we may remark that, after they emerge from the inguinal canal, they appear in the spermatic cord, surrounded and enveloped by the spermatic veins. When the artery reaches from one to three inches from the epididymis, it divides into two branches, which descend to the testicle on its inner side, opposite to that on which the epididymis is placed. One passes to the anterior and upper—the other to the posterior and lower part of the testis. From the former the vessels of the epididymis arise; one passing to its caput, another to its body, and a third to its cauda and the first convolutions of the vas deferens, communicating freely with the artery of the vas deferens. The spermatic artery then enters the testis, by penetrating the outer layer of the tunica albuginea; then, dividing upon its vascular layer, its branches form an arch by their junction at the lower part of the testis, from which numerous vessels pass upwards, and again descending, supply the lobes of the tubuli seminiferi. Besides this lower arch, there is another, passing in the direction of the rete, extremely convoluted in its course, and forming an anastomosis between the principal branches.

*Of the Spermatic Veins.* We may pass over the peculiarities of their abdominal distribution, as they are familiar. Two or three veins often accompany each spermatic artery in the abdomen, and similar branches also cross upon the coats of the artery, and form several anastomoses; but they unite into one before they terminate. In the inguinal canal, they are placed with the spermatic artery, but are divided into two, three, or more vessels, beside some small communicating branches. Below the external abdominal ring, they present the following distribution.

Three sets spring from the testis; one from the rete and tubuli, another from the vascular layer of the tunica albuginea, and a third from the lower extremity of the vas deferens. The veins of the testis pass in three courses into the beginning of the spermatic cord; two of these quit the back of the testis, one at its anterior and upper part, and a second at its centre, and after passing from two to three inches, these become united into one. The other column accompanies the vas deferens. There is also a large vein just above the testis, which crosses to join the three columns. The veins of the epididymis are—one from the caput, another from its body, one from its cauda, and another from its junction with the vas deferens, besides some small branches; they terminate in the veins of the cord. The veins of the cord below the external ring divide into numerous branches, which are not only turned and twisted upon each other, but very frequently communicate; so that, although they have valves, they may be injected contrary to the course of the blood, by the injection traversing from one to the other. These vessels have been absurdly called the vasa pampiniformia.

The length and size of the spermatic arteries and veins, and their numerous convolutions, seem to render it obvious that Nature has designed the circulation to be slow, the secretion elaborate, and that she has defended the tender structure of the testis from the danger of an impetuous current. The number and size of the spermatic veins, when compared to their arteries, are calculated to secure the same end.

There is a second artery in the cord, which begins from the vesical arteries, near the remains of the umbilical. This deferential artery divides into two sets of branches: one descending to the vesicula seminalis and to the termination of the vas deferens; the other ascending upon the vas deferens, runs in a serpentine direction upon its coat, passes through the whole length of the spermatic cord, and when it reaches the cauda epididymis, divides into two sets of branches—one advancing and uniting with the spermatic artery to supply the testicle and epididymis—the other passing backwards to the tunica vaginalis and cremaster.

### ABSORBENT VESSELS OF THE TESTICLE.

These arise both from the coats of the testis and from its internal structure. They unite upon the cord, and form three or four trunks, which ascend on the spermatic veins, pass through the inguinal canal, and diminish in number but increase in size when they enter the cavity of the abdomen. On the right side they quit the spermatic vein to cross the vena cava, and terminate in three or four absorbent glands by the side of the aorta, near the origin of the spermatic artery. On the left side they pass into glands in contact with the aorta, just below the renal artery. The absorbents of the tunica vaginalis terminate in those of the testis.

From the description of the INGUINAL CANAL, and a very good one it is, we shall only cull one passage. The cremaster muscle is connected with the muscular fibres both of the internal oblique and the transversalis. But it is more particularly the latter to which we would direct attention. The transversalis muscle, then, forms an arch over the spermatic cord, and is inserted, with the tendon of the internal oblique, into the tendinous covering of the rectus. But its lower edge has a very peculiar insertion. It begins to be fixed into Poupart's ligament, almost immediately below the commencement of the internal ring, and it continues to be inserted behind the cord into Poupart's ligament, as far as the attachment of the rectus. Thus the inguinal canal is endowed with muscular contraction, which, during the action of the abdominal muscles, serves to close it, and lessen the disposition to hernia. Sometimes a portion of muscle descends from the tendon of the transversalis in the course of the linea semilunaris, to be inserted into the fascia transversalis behind the cord, and into Poupart's ligament. It is this circular insertion of the transversalis, which, according to Sir Astley, is the cause of stricture in inguinal hernia, in the course of the canal and nearly at the upper ring. This will be new to most anatomists; of its correctness we have satisfied ourselves by several dissections.

### OF THE CORD BELOW THE EXTERNAL RING.

The account of the anatomy of the spermatic cord in this situation is too valuable to be passed unnoticed. It is loosely covered by a superficial fascia, continuous with that of the abdomen and of the scrotum, but adhering strongly to the edges of the external ring. Of this we need say no more. The second covering of the cord is the cremaster muscle. It arises from Poupart's ligament in the inguinal canal, between the internal oblique and the transversalis, blending there with some of the fibres of those muscles; below the origin of the fibres of the internal oblique, it arises from

Poupart's ligament nearly to the external ring; behind the spermatic cord it receives muscular fibres from the transversalis; it is also attached, on the inner side of the abdominal ring, to the lower part of the sheath of the rectus muscle. From these attachments it descends upon the cord in loops. It envelops the vessels and nerves of the cord in its descent, and forms numerous tendons, which resemble in their first appearance nervous filaments. It has the following insertions:—first, it forms a tendinous sling, which envelops the lower part of the tunica vaginalis; secondly, it sends tendinous fibres into the inferior part of the testis and epididymis, and into the tunica vaginalis; and thirdly, it blends with some cords which surround and enclose the lower part of the vas deferens, and which may be traced to the upper orifice of the inguinal canal, and which pass down with the spermatic vessels.

The cremaster has an artery to supply it, which is the third artery of the cord. This cremasteric artery arises from the epigastric, near the internal opening of the inguinal canal; passes inwards towards the lower part of the rectus and pyriformis muscles, nearly in the line of Poupart's ligament internally, and then divides into two branches:—the first passes to the rectus and pyriformis muscles; the second descends upon the back part of the cord to the cremaster, to which it gives vessels in its course. The vein accompanying this artery terminates in the epigastric vein, and a branch of a nerve attends them. The use of the cremaster is to draw up the testis *in coitu*, pressing it against the pubes and abdominal ring, and thus aiding the passage of the semen as it is secreted. When examined in a full-grown fœtus, it appears that the testis has been drawn down into it, as into a purse; and if the testis has not long descended, and its adhesions to it are slight, it can be easily drawn from the cord and testis, excepting at its lower part, where it firmly adheres to the tunica vaginalis reflexa, and to the remains of the gubernaculum, the epididymis, testis, and vas deferens.

### Of the Descent of the Testis.

The testes in the first seven to eight months of fœtal life are found situated on the loins. It is only in the earliest months that they are placed immediately below the kidneys; in a fœtus of five to six months they are seated on the lower part of the psoæ muscles. Like the other abdominal viscera the testis is covered by peritoneum on its fore part and sides; this forms the tunica vaginalis testis of the adult. From the lower end of the testis and epididymis the gubernaculum proceeds, behind the peritoneum, but covered by it on its fore part and sides. It is composed of several strong ligamentous fibres, which proceed through the inguinal canal to the cellular membrane of the scrotum in which it is lost. The peritoneum of the lower part of the abdomen passes down upon, and adheres to the gubernaculum, so as to form a small pouch in the inguinal canal, to which the cremaster is attached. The vas deferens descends behind the peritoneum, from the lower end of the epididymis, passing posteriorly to the gubernaculum over the psoas muscle and iliac vessels to the duct of the vesicula seminalis behind the bladder, which are so little buried in the pelvis that the latter can be brought into view without dissection. The cremaster, as far as Sir Astley can distinguish it in the fœtus, passes upon the gubernaculum to the testis and epididymis, and is attached to the process of peritoneum which descends with the testis as a pouch, to the lower part of the inguinal canal; and the testis descends into this muscle as into a purse, as it is directed down by the gubernaculum, whence the loops which it forms.

"If any one will be at the trouble to examine a fœtus at the eighth or ninth month, soon after the testis has descended, he will find that the cremaster may be readily turned from the spermatic vessels and vas deferens, so as to leave them free from it; and it can be separated from the epididymis and testis, excepting at the lower extremity of each of those bodies, and the lower end of the vas deferens, into which it is inserted, so that it forms a purse to the testis and cord, after their descent.

In animals, in whom the testis remains in the abdomen, the cremaster still exists. I do not believe that it is the cause of the descent of the testis, nor that it is designed as a suspendor, but as a compressor of the testis.

I will merely put it as a query—if the descent of the testis may not be assisted by the pressure of the fluid, provided in the abdomen of the fœtus to allow of the growth of parts, upon the pouch of the peritoneum, which adheres to the gubernaculum, and which assists in forming the tunica vaginalis reflexa?" 43.

If the testis has not descended at birth, it is often afterwards forced down by a congenital hydrocele or hernia. The descent of the testis begins at the very earliest period of its formation, and it usually reaches the scrotum about the eighth month, though it varies greatly in point of time. The peritoneum attached to the gubernaculum, and the loose peritoneum lining the lower part of the abdomen, descend with the testis between the eighth and ninth months; for the testis is not drawn into the pouch, but the testis, pouch, and loose peritoneum of the lower part of the abdomen descend together. The peritoneum attached to the gubernaculum becomes the tunica vaginalis reflexa of the adult; the portion covering the testis in the abdomen is the tunica vaginalis testis in man; and that which it draws after it from the abdomen to the testis is the tunica vaginalis of the cord. Very soon after the descent of the testes the peritoneum becomes closed by the process of adhesion, first towards the abdomen, then gradually lower down; the exact time of its being shut is uncertain. At the ninth month Sir Astley has often found both open; or one open, and the other closed. The peritoneum becomes shut from the abdomen nearly to the testis.

The time at which the testis descends varies greatly in different persons. It usually reaches the scrotum before the birth of the infant; but frequently one is placed in the scrotum, and the other remains in the abdomen, or in the inguinal canal, just above the external ring, or sometimes it just emerges from the ring. In these situations it is exposed to injury and violence; and if it remain, it is rather prone to disease of a malignant character. Sir Astley has often seen the testicle descending between the age of thirteen and seventeen, probably from some new excitement about that period; the descent is, in some cases, not accomplished till the age of twenty-one. When the testis remains in the abdomen the mind of the patient is affected with the suspicion that his virility is lessened or destroyed; one such individual committed suicide. In this case, and in others which Sir A. has examined, the testis was nearly of the same size as a healthy testis deprived of its tunica vaginalis, and the seminiferous tubes were full of semen.

It often happens that when the testis remains in the inguinal canal, there are severe spasms of the cremaster, or muscles of that canal, accompanied with violent pain, and only relieved by the hot-bath and fomentation. The tunica vaginalis is generally closed at birth, but it often is open on one side, sometimes on both. If the opening be so small as only to admit serum, a congenital hydrocele is produced. A truss applied in infancy cures this disease; the water being absorbed when the tunica vaginalis is closed.

The opening in the tunica vaginalis is sometimes partially closed, and produces hydrocele of the spermatic cord; this, however, is also the result of serous cysts forming in the cord, more especially just above the testis. The opening in the tunica vaginalis sometimes remains small until the adult age, and then it becomes suddenly dilated by the protrusion of intestine, forming a hernia congenita; and when the surgeon in the operation discovers its nature, the patient assures him he never had hernia until a few days before. This Sir A. has known occur several times. More frequently the tunica vaginalis, when unclosed, admits protrusion of the intestine in childhood, in contact with the testis, constituting hernia congenita. When the testicle has not descended at birth it often happens that a hernia becomes the means of its descent; and such hernia should remain without a truss being applied, until it has brought down the testis into the scrotum. A testis late in its descent and protruded by hernia is often lessened in its bulk; but the testis on the other side, with this diminished organ, is sufficient for the procreation of children. The tunica vaginalis is sometimes closed by a film of adhesion; which, becoming elongated by intestinal protrusion, forms a sac, in the mouth of which the intestine has been strangulated; and the patient dies, if unrelieved by an operation.

### OF THE NERVES OF THE TESTIS, AND CORD, AND PARTS ADJACENT.

Three sets of nerves supply the testis and neighbouring parts: the first are those in the vicinity of the external ring; the second, the external spermatic nerves distributed to the cord; and the third, the spermatic plexus, derived from the grand sympathetic. We may cursorily mention that the first is derived from a muscular branch, which winds round the crest of the ilium, and may be traced back to the first and second lumbar nerves—that the second, or external spermatic nerve, is derived from the second lumbar nerve, and pierces the psoas muscle—and thirdly, that the spermatic or ganglionic plexus may be considered as consisting of two portions, the first descending, along with the spermatic vessels, from branches of the superior mesenteric, renal, and aortic plexus, the second being derived from the hypogastric plexus.

If the peritoneum at the internal ring be examined, it will be found firmly united by tendinous cords to the fascia transversalis. These cords descending with the vas deferens form a sheath to it, and, passing from one convolution to the other, preserve it in its convoluted state; they terminate in being fixed in the cauda epididymis, and lower extremity of the testis, blending there with the cremaster. In the same manner cords pass down with the spermatic artery, and form a sheath, by which it is enveloped, to preserve its convolutions. To discover these cords, it is only necessary to dissect closely on the coats of the vas deferens and spermatic artery, especially below the external ring.

The testis in youth is capable of being injected. At two years the vas deferens, epididymis, vasa efferentia, and rete exist; but the tubuli are imperfect, or are too small to receive injection. In advanced age the testis becomes reduced and relaxed, from the diminished size of the seminiferous tubes, and from the smaller quantity of fluid which they contain. It is common, in advanced age, to find the caput epididymis diseased; several of the lobes of the vasa efferentia being converted into a yellowish-brown solid structure. In age the seminiferous tubes become small; they appear yellow instead of red, from their having less arterial blood; and it often happens that a considerable number of them become cords instead of tubes, assuming a ligamentous appearance. A varicose state of the left testis is frequent in age.

The testis does not in general become absorbed, if it be partially diseased, although its functions may be interrupted even to complete obstruction of semen. In 1823 Sir Astley divided the vas deferens upon one side in a dog, and the spermatic artery and vein on the other. The testis on the side upon which the artery and vein were divided died and sloughed away. On the side upon which the duct was divided the testicle became somewhat larger than natural. During six years the dog was twice seen *in coitu*, but the female did not produce. In 1829, Sir Astley killed him, and found the vas deferens below the division excessively enlarged and full of semen, and entirely obstructed, with some separation of its extremities; but it was open from the place of division to the urethra. Some time ago it was attempted in one of the Borough hospitals to cure malignant disease of the testicle by removal of part of the vas deferens, on the supposition that this would occasion wasting of the organ. We ridiculed the idea, and conjectured that such an operation would be more likely to occasion repletion than depletion of the testis; a conjecture confirmed by Sir Astley's experiment.

The wasting of one testis in early life does not prevent the person from having children afterwards. Neither does the removal of one testis seriously diminish the virile powers. A gentleman had a testis removed in January, 1821, for an enlargement and great hardness. He recovered in three weeks. His wife, by whom he had already had one child, nursed him during his confinement, and in March she proved pregnant, about nine weeks after the performance of the operation. A man whose testis had been absorbed for fourteen years, by wearing a truss for congenital hernia, has since married and has now a child not quite a year old.

It has twice fallen to Sir Astley's lot to remove the testis of persons who had already lost one. In 1799 Mr. Cooper removed the testis of a man at Guy's. This and the following are related in Sir Astley's characteristic manner, and we cannot mutilate them.

"The second operation (on the patient alluded to) was performed by myself in Guy's Hospital, in June 1801, for a chronic abscess in the testis. On visiting him four days after the operation, he informed me that he had, during the last night, an emission, which appeared upon his linen; and, struck with the curiosity of this circumstance, I requested my then apprentice, Mr. Travers, to occasionally visit him after his recovery, and he had quitted the hospital; and I have myself, during the twenty-nine years which have since elapsed, repeatedly seen him. He had been married prior to the loss of one testis.

For nearly the first twelve months, he stated that he had emissions *in coitu*, or that he had the sensations of emission. That then he had erections and coitus at distant intervals, but without the sensation of emission. After two years he had erections very rarely and very imperfectly, and they generally immediately ceased under an attempt at coitus.

Ten years after the operation, he said he had during the past year been once connected.

In 1829 he visited me, because he was a severe sufferer from piles. He then stated that for years he had seldom any erection, and then that it was imperfect; that he had no emissions from the first year of the operation; that he had for many years only a few times attempted coitus, but unsuccessfully; that he had once or twice dreams of desire, and a sensation of emission, but without the slightest appearance of it. The penis is shrivelled and wasted. He shaves once a week, and sometimes twice. His voice, naturally rather feeble, remains as at the time of the operation.

From this man's declarations, I believe that the history of eunuchs, if perfectly castrated, has been very much misrepresented; for it would seem that, after a few months, he lost all seminal emission, but that the erectile power remained for a few months more; and then, excepting at very distant periods, and but imperfectly, even that power ceased, and the penis became shrivelled and diminished.

The second case in which I removed the testis, was in a lad in Guy's Hospital, aged sixteen years, who had previously the other testis extirpated. The disease each time was a scrofulous abscess, with subsequent ulceration. The lad had not reached puberty, and he was very weakly and emaciated. Five years afterwards, as I was stepping out of my carriage at a patient's door, a fat sleek-looking young person said—'how do you do, sir?'—I said, 'very well, but I do not know you.'—'Have you forgotten removing my testicle in Guy's Hospital, five years ago?' 'Oh yes, I recollect you; you look very well.'—'Yes, but I am very unhappy;' and he immediately burst into tears.—'Why, what do you lament?'—'Oh sir, that I am not as other men—I often wish that I were dead.' Desirous to cheer him, I said—'you are a lucky fellow, for you are saved from many evils.'—He shook his head, and I left him sorrowful." 55.

This concludes the anatomical portion of Sir Astley Cooper's volume. The other parts have been copiously analyzed before, and it now only remains for us to take leave of the worthy Baronet. Our readers are well aware that it is not our custom to notice elementary matter, and therefore we seldom or never review those multifarious and often very excellent treatises on anatomy, physiology, &c. which are daily issuing from the press. But we cannot look upon even the anatomical part of this work in such a light. The descriptions are so excellent, frequently so original, always so correct, that we should not consider ourselves justified if we did not give them currency in the pages of a journal which professes to offer useful information to its readers. Without this part the practical doctrines would be only partially understood, and certain we are that no well-informed man in the profession will quarrel with us for what we have done. The majority of medical men could but ill afford, indeed they could not afford at all, to purchase so expensive a work, and to them it must be acceptable to receive the substance of the whole through the cheap medium of a periodical publication. So much for ourselves and now a word to the author on parting. He has done himself honour by this work, he has shewn that in age he lacks little of the energy, and none of the zeal of his youth and his manhood. With his usual penetration he has seen the road to fame and to honour, and has pursued it with his usual straightforward determination. Long may we boast of him amongst us as of one of our greatest ornaments; long may he retain the wish and the power to convey information to his brethren; in the words of the lyric bard, though without intending quite so gross a compliment:—

Serus in cœlum redeat, diuque

Lætus intersit populo!

---

